# Brain correlates of action word memory revealed by fMRI

**DOI:** 10.1038/s41598-022-19416-w

**Published:** 2022-09-26

**Authors:** Zubaida Shebani, Francesca Carota, Olaf Hauk, James B. Rowe, Lawrence W. Barsalou, Rosario Tomasello, Friedemann Pulvermüller

**Affiliations:** 1 Cognition and Brain Sciences Unit, University of Cambridge, 15 Chaucer Road, Cambridge, CB2 7EF UK; 2 Psychology Department, Sultan Qaboos University, Muscat, Oman; 3 Max-Planck Institute for Psycholinguistics, Wundtlaan 1, Nijmegen, The Netherlands; 4 Brain Language Laboratory, Department of Philosophy, Freie Universität Berlin, 14195 Berlin, Germany; 5 Berlin School of Mind and Brain, Humboldt Universität zu Berlin, Berlin, Germany; 6 Department of Clinical Neurosciences and Cambridge University Hospitals NHS Trust, Cambridge University, Cambridge, CB2 2QQ UK; 7 Institute of Neuroscience and Psychology, University of Glasgow, Glasgow, UK; 8 Cluster of Excellence ‘Matters of Activity. Image Space Material’, Humboldt Universität zu Berlin, 10099 Berlin, Germany

**Keywords:** Psychology, Neuroscience, Cognitive neuroscience, Computational neuroscience

## Abstract

Understanding language semantically related to actions activates the motor cortex. This activation is sensitive to semantic information such as the body part used to perform the action (e.g. arm-/leg-related action words). Additionally, motor movements of the hands/feet can have a causal effect on memory maintenance of action words, suggesting that the involvement of motor systems extends to working memory. This study examined brain correlates of verbal memory load for action-related words using event-related fMRI. Seventeen participants saw either four identical or four different words from the same category (arm-/leg-related action words) then performed a nonmatching-to-sample task. Results show that verbal memory maintenance in the high-load condition produced greater activation in left premotor and supplementary motor cortex, along with posterior-parietal areas, indicating that verbal memory circuits for action-related words include the cortical action system. Somatotopic memory load effects of arm- and leg-related words were observed, but only at more anterior cortical regions than was found in earlier studies employing passive reading tasks. These findings support a neurocomputational model of distributed action-perception circuits (APCs), according to which language understanding is manifest as full ignition of APCs, whereas working memory is realized as reverberant activity receding to multimodal prefrontal and lateral temporal areas.

## Introduction

When reading and listening to action words, we automatically think of the respective action. This recognition of action words is accompanied by the instantaneous neurophysiological activation of motor systems^1–7^. The reverse functional link between action and language systems is shown by behavioural and TMS studies in which motor system activity modulates the processing of action words^8–14^. For example, stimulating the motor cortex using TMS modulates the recognition of semantically-specific types of action words^8,10^ and motor movement can interfere with or facilitate action word processing and memory^11,15–17^ (but see^18^) just as the processing of action related words and sentences can interfere with or assist motor movement^19,20^. Additionally, dysfunction of motor systems found with focal cortical damage or more widespread progressive disease impairs the processing of action words and concepts^21–26^, but see^27^ for different results in a case study of a patient with impaired action execution and see^28^ for results implicating posterior temporal cortex in action representation.

Together, these results suggest a causal meaning-dependent influence of motor systems on action language processing and lead to the hypothesis that a network of interacting areas contributes to both action-semantics and symbolic-linguistic processes for the perception and comprehension of action-related words; the contribution of motor areas has been proposed to be crucial because it provides the necessary semantic grounding of the linguistic symbols in bodily action^1,29^ (for alternative views, see^30,31^). The same network of interacting areas involved in action word perception and comprehension has been suggested to also be relevant and critical for the semantic maintenance of action words in working memory.

Working memory refers to the retention and processing of information that is just experienced but no longer available in the external environment, or to information retrieved from long-term memory^32,33^. Over the past 30 years, several cognitive models of working memory have been proposed, the most influential of which is Baddeley’s model of working memory (e.g.^34,35^). This model includes a ‘central executive’ to control attention and to manage information in verbal and visuospatial buffers. Internal representations held in working memory can be actively maintained through rehearsal strategies mediated by sub-vocal articulation. Verbal working memory engages a network of brain regions thought to be involved in articulatory and auditory phonological processing, including inferior frontal (Broca’s area) and superior temporal cortex (Wernicke’s area) along with parietal cortex^36–38^. A key region associated with verbal memory tasks is the dorsolateral prefrontal cortex (PFC), which is sometimes presented as the locus of the ‘frontal executive’ functions^35^. More recent models of working memory have suggested an additional mechanism, attentional refreshing, through which verbal stimuli can be maintained^39–42^. This attention-based mechanism is thought to refresh semantic representations of words, operating in conjunction with but independently from articulatory rehearsal to sub-serve working memory^43–46^. Attentional refreshing has also been associated with activity in the left dorsolateral PFC^47,48^. Different areas of PFC have been found to support different memory sub-functions (memory maintenance to BA 9 and memory-response selection to BA 46,^49^). Convergent input to this region from sensory, motor areas and association cortex may explain why this region plays such a prominent role in memory processes^50,51^. In this sense, working memory may not just be the product of the PFC but of its interactions with posterior cortical areas^32,33,52–54^.

The neurobiological mechanisms of working memory have been elucidated by intracortical recordings. From this research, it emerged that neurons in prefrontal cortex and in multimodal parietal and temporal regions are most likely to include ‘memory cells’ indicating specific content which supports working memory processes. Interestingly, parallel cell dynamics and memory-content specificities were found in frontal and temporal systems and temporary lesions in one of these systems were observed to entail functional changes in the other (for review, see^33,53,55^). This body of evidence enforces the position that it is not areas that are responsible for working memory but neuronal ensembles, called action perception circuits (APCs), whose strongly interlinked neuron members are distributed across several areas and maintain their reverberant activity for some time after full activation or ‘ignition’.

**Figure 1:**
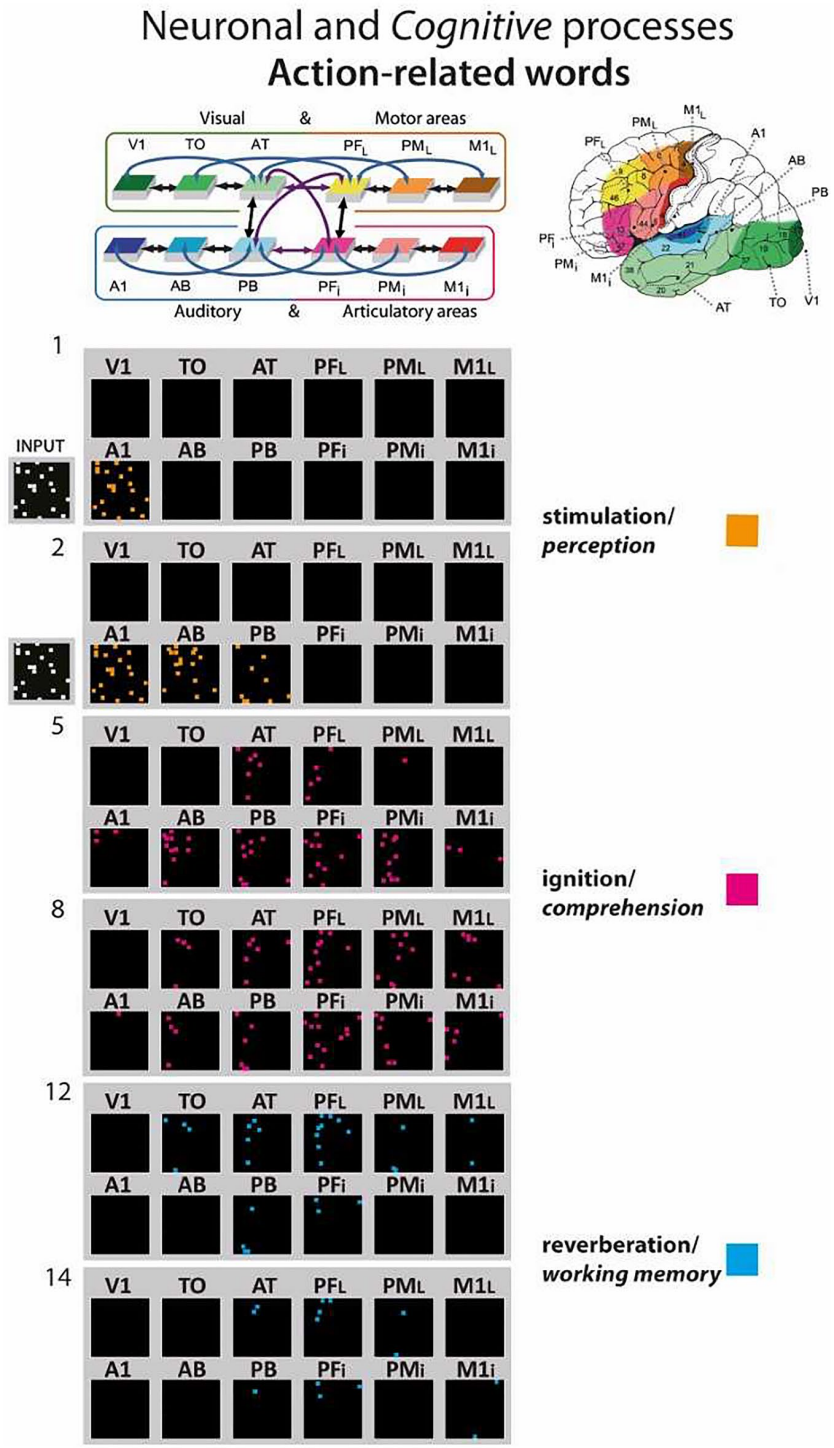
Simulated action word processing in a biologically constrained spiking network model of the fronto-temporo-occipital lobes. After the network underwent action word learning by interlinking acoustic, articulatory and action-semantic information, the action-word-related circuit was re-activated by auditory stimulation to areas A1(word recognition). The re-activation process comes in different consecutive neuronal and cognitive phases, the stimulation phase, which corresponds to word perception (orange pixel), the full activation or ‘ignition’ phase, the correlate of word comprehension (magenta pixel), and the reverberant maintenance of activity, which underpins verbal working memory (blue pixels). Please note the relatively prominent role of prefrontal cortex in the reverberation and working memory phase, which motivates the prediction of an anterior frontal activity shift. At the top right, the 12 brain areas modelled are shown. The top left box-and-arrow diagram shows the structure of the network; box colours and positions indicate correspondence to brain area and arrows between area connectivity. Sets of 12 black squares in the main diagram below represent activation of the same 12 areas at a given simulation time step. Simulation time steps are indicated on the left. Each coloured dot represents one active (spiking) model neuron at a given time step. Figure adapted from Tomasello et al.^60^.

Neurocomputational modelling of word learning show the emergence of action perception circuits distributed across language areas^56,57^. These neuronal circuit models explain why language and symbol processing activate classic language areas along with multimodal prefrontal and temporal areas, and even, depending on semantic word type, additional category-preferential areas such as the motor cortex^58–61^. Tomasello et al.^60^ simulated word-learning processes, as documented by language developmental studies (e.g.,^62^), in a frontal-temporal-occipital brain model constrained by connectivity structures at the global and local scales (for more detail about the biological constrain of the model see also^63^). The model equipped with unsupervised Hebbian learning gave rise to realistic activations of conceptual and semantic circuits across multiple cortical regions and made critical predictions on how neuronal circuit dynamics change over time. Upon brief stimulation of the circuit, the model showed an initial perceptual activation phase related to acoustic/visual perception (orange pixels, Fig. 1) followed by a full ‘ignition’^64–66^ involving the entire distributed circuit corresponding to word recognition and comprehension (magenta pixels). These spatiotemporal dynamics correspond the experimentally measured near-simultaneous activations revealed by MEG recordings from superior temporal and frontal areas in the perisylvian system, and slightly later, in dorsal motor cortices, which were observed during action-related word processing^67,68^. Importantly, the model makes critical predictions about the activation dynamics of verbal working memory by showing that, after full activation or ‘ignition’^64–66^ involving the entire distributed circuit, activity persists for a while during a ‘reverberation period’. Following established biological theories of working memory^33,53^, this maintained activity within the circuit can be interpreted as a correlate of verbal working memory (blue pixels, Fig. 1) after which activity disappears due to neuronal inhibition and fatigue. Critically, only neurons in those parts of the network most strongly interlinked with other circuit members are able to maintain activity over several seconds and thus contribute to working memory. These neurons are primarily located in multimodal areas with high degree of connectivity (so-called ‘connector hubs’) where information from different modalities converge, which are considered to play a special role in cognition^69^. Therefore, after ignition, activity retreats from the modality-preferential areas relevant for grounding to multimodal connector hubs. As shown in Fig. 1, in the frontal cortex, this would result in an anterior shift from motor cortex to adjacent frontal and prefrontal cortex^57,59^. In contrast to this prefrontal memory perspective, a strong version of an embodied perspective on semantic meaning may put that, similar to symbolic understanding, the memory maintenance of action words draws primarily on motor systems. Whereas the involvement of motor systems in passive recognition of action verbs has been supported by some studies (see^57,70–73^), other studies using nouns referring to *objects* that afford actions (e.g. a wrench affords the action ‘grasp’) did not find a role of motor systems in working memory^74,75^.

In the present study, we used arm and leg related action words to examine brain correlates of verbal working memory. A low load condition with four repetitions of the same to-be-memorised action word was compared with a high load condition with four different words that are semantically closely related. This high load versus low load contrast was used as it ensured that stimuli in the two conditions were similar and that conditions differed essentially in memory load. Hemodynamic responses were obtained when subjects read and encoded word stimuli and subsequently when they maintained them in their working memory. As the active maintenance of four different and semantically related words may draw more heavily on neural resources than the active maintenance of a single word (repeated four times), we expected stronger activation during the high load memory maintenance period compared with the low load condition in one of three possible brain loci: (1) motor regions previously found active during the perceptual processing of action words, as predicted by embodied theories, (2) areas involved in verbal working memory including Broca’s area, as predicted by the Baddeley model, or (3) frontal areas anterior to the motor regions previously found active during action word perception, as predicted by recent neurocomputational modelling (i.e., anterior shift). Furthermore, we asked whether semantic differences between word types, namely their respective relationship to upper and lower extremities, might lead to category-specific activations reminiscent of the semantic somatotopy found in passive word reading or recognition experiments.

## Methods

### Participants

Nineteen monolingual, native English speakers participated in the study. All were right handed with an average laterality quotient^76^ of 74.9% (s.d. = 22.6). All gave their written, informed consent and were reimbursed for their time. One subject was discarded prior to statistical analysis of fMRI data due to excessive movement during the acquisitions (more than 10 mm). A further subject was discarded due to poor performance on the behavioural task (50% errors). Therefore, data from 17 subjects (9 male; aged 21–35, mean 25.5, SD 3.8) are reported below. All participants had normal or corrected-to-normal vision, were without psychiatric or neurological illnesses and did not use any medication or drugs. Ethics permission was obtained from the Cambridge Psychology Research Ethics Committee and all methods were performed in accordance with the relevant guidelines and regulations.

### Stimuli

Lexical stimuli for the task consisted of 80 action words, 40 semantically related to the arm (e.g., ‘pick’, ‘grasp’) and 40 to the leg (‘walk’, ‘kick’). They were matched for several psycholinguistic variables (see Table 1) including word, lemma, bigram, and trigram frequencies, and their number of letters and phonemes. Lexical stimuli were also matched for grammatical ambiguity, and for ratings of valence, arousal, imageability, visual relatedness, body-relatedness and general action-relatedness as revealed by previous semantic ratings^5^. In addition to the original set of 80 arm/leg words, 10 action words (5 arm/5 leg) were used as probes in non-match trials. Each of these probe words was used in only 3 or 4 non-match trials. Two different pseudo-randomised stimulus sequences with the same repetition structure for arm and leg words were used and alternated between subjects.

**Table 1:** Means and standard errors of psycholinguistic and semantic properties for arm and leg words.

Variable	Arm words		Leg words	
	Mean	SE	Mean	SE
Number of phonemes	3.73	(0.12)	3.90	(0.14)
Number of letters	4.45	(0.14)	4.57	(0.13)
Grammatical ambiguity	1.93	(0.04)	1.95	(0.03)
Word frequency	219.8	(47.0)	232.8	(48.2)
Lemma frequency	520.2	(82.1)	540.5	(89.9)
Bigram frequency	30,196	(2506)	34,859	(2726)
Trigram frequency	3250	(386.4)	3076	(317.2)
Valence	3.65	(0.14)	3.96	(0.14)
Arousal	3.04	(0.14)	3.12	(0.16)
Imageability	4.60	(0.12)	4.53	(0.14)
Visual relatedness	4.40	(0.16)	4.14	(0.16)
Body relatedness	3.71	(0.16)	3.74	(0.14)
Action relatedness	5.06	(0.14)	5.11	(0.17)

### Procedure

During MRI scanning, subjects performed a delayed non-match-to-sample task consisting of four blocks. In each trial, subjects were presented with either four arm- or leg-related action words. Sample words were presented serially for 200 ms each. The stimulus onset asynchrony of two subsequent words was 500 ms (two words per second). Stimuli presentation was followed by a memory period during which subjects were required to keep the four sample words in memory. The length of the memory period varied between 4 and 14 s. Six durations were used (4 s, 6 s, 8 s, 10 s, 12 s and 14 s) which were randomized across trials. After the delay, the probe word (from the same word category) was shown for 1 s. Subjects were required to press a button with their left index finger whenever the probe word was *different* from all of the four previous words in that trial; subjects were asked not to do anything when probe stimuli matched one of the four sample words. Variable delay periods, including long memory delays, were used to allow the disambiguation of the fMRI responses to the stimuli from those recorded during memory maintenance. In order to minimize movements in the scanner, subjects were instructed to respond by button press in non-match trials only, which constituted 20% of all trials. Left hand responses were required to minimize motor-related activation in the left language-dominant hemisphere, where relevant language-related activations were expected. Subjects were instructed to respond to the probe as fast and as accurately as possible and had up to 4 s to respond in each trial. Note that probe words in non-match trials were different arm/leg-related action words than those in the original set of 80 action words and never presented in the task as sample stimuli. A variable-length inter-trial interval (8–12 s, counterbalanced) separated all trials.

Memory load was varied between trials. In the high-load condition, four different action words, which were very close in meaning, were presented, whereas in the low-load condition a single action word was shown four times. Each block consisted of 40 trials, 20 with arm- and 20 with leg-related action words, each group again subdivided into 10 high- and 10 low-load trials. Trials were pseudo-randomised within each block so that not more than two trials of the same action word category (arm/leg) appeared consecutively.

Before scanning, subjects viewed task instructions and performed a practice version of the task. Responses and reaction times were recorded using an MRI compatible button box. The task was designed and presented and behavioural data was recorded using E-Prime 1.1 (Psychology Software Tools Inc., Sharpsburg, BA, USA).

### fMRI data acquisition

Participants were scanned on a Siemens (Erlangen, Germany) TIM Trio 3 T machine at the MRC Cognition and Brain Sciences Unit (MRC-CBSU), Cambridge, UK. The nonmatching-to-sample task was performed during 4 separate sessions of echoplanar imaging (EPI) with 460 volumes acquired in each session (including 12 s of initial dummy scans to allow steady state magnetisation). Acquisition parameters used were as follows: TR = 2.02 s; TE = 30 ms; flip angle = 78°. Functional images consisted of 32 interleaved slices covering the whole brain (slice thickness 3 mm; matrix size 64 × 64; interslice gap 25%; in-plane resolution 3 × 3 mm; see http://imaging.mrc-cbu.cam.ac.uk/imaging/ImagingSequences). Stimuli were back-projected onto a screen using a Christie video projector with a 60-Hz refresh rate, and viewed using a mirror mounted on the head-coil. Soft padding minimized head movement during the scanning session.

### fMRI data analysis

Imaging data were processed and analysed using the SPM5 (Wellcome Department of Imaging Neuroscience, London, UK; http://www.fil.ion.ucl.ac.uk/spm/). Images were motion corrected and slice time corrected then realigned to the first image using sinc interpolation. Any non-brain tissue (e.g. skin, fat, muscle) was removed from the T1-weighted structural images using a surface model approach^77^. The EPI images were coregistered to these structural T1-images using a mutual information coregistration procedure^78^. The structural MRI was then normalized to the 152-subject T1 template of the Montreal Neurological Institute (MNI). The resulting transformation parameters were applied to the coregistered EPI images. During spatial normalization, images were re-sampled with a spatial resolution of 2 × 2 × 2 mm^3^ and spatially smoothed with a 10 mm full-width half-maximum Gaussian kernel. Preprocessing was automated using in-house software (http://imaging.mrc-cbu.cam.ac.uk/imaging/AutomaticAnalysisManual).

Individual subject activations were analysed using a general linear model approach^79^. A high-pass filter was used to remove low-frequency noise in the signal (cutoff period 128 s). The data for each subject were modelled using a boxcar design convolved with the canonical haemodynamic response function. Events of interest and time points modelled were as follows: encoding (0–2 s), memory period (2–11 s ± 5 s, adjusted to the length of individual memory periods) and two probe events at the end of the memory period, one for trials requiring a response and one for non-response trials. This generated a time-course of predicted neural activity for each event type allowing us to estimate changes in haemodynamic signal for arm/leg word stimuli in the high and low load memory conditions. Four stimulus events (hi/lo memory load condition, arm/leg words) were distinguished in the encoding and memory maintenance intervals respectively; additional response and non-response events were coded for the final retrieval interval. Contrasts were run to estimate signal changes associated with these events at each voxel and the resulting maps from each subject were entered into a second level (group) analysis treating subjects as a random variable. Brain activations are displayed after controlling for false discovery rate (FDR) at 0.05 for multiple comparisons. Stereotaxic coordinates for voxels with maximal z values within activation clusters are reported in MNI standard space. Anatomical labels of nearest cortical grey matter for peak coordinates were obtained from the MRIcron software (http://www.sph.sc.edu/comd/rorden/mricro.html), based on the anatomical parcellation of the MNI brain published by^80^.

### ROI analyses

In addition to the whole brain analysis described above, two analyses were performed to examine activity in regions of interest (ROI). The first of these (hereinafter ROI Analysis 1) focused on activation differences between the initial memory encoding interval and the subsequent memory maintenance epoch and compared memory load effects for arm and leg related action words. For data driven ROI definition, clusters activated due to memory load (encoding and maintenance periods together) using a whole-brain corrected significance criterion were used. Each absolute activation maximum (that is, the voxel with the highest t-value in its respective significant cluster) was defined as the centre of an ROI with radius 10 mm. The MarsBar software utility (http://marsbar.sourceforge.net/) was used to average parameter estimates over voxels and to estimate signal changes in these regions for each time interval (encoding, maintenance), word type (arm, leg) and subject. These data were then submitted to a repeated measures analysis of variance (ANOVA). F-Tests, Bonferroni-corrected for multiple comparisons, were used as planned comparison tests.

The second ROI analysis (hereinafter ROI Analysis 2) was performed in order to examine whether working memory produces activation in regions anterior to those found for action word perception. Four ROIs (two lateral and two dorsal precentral) were selected based on local activation maxima in frontocentral sensorimotor cortex during encoding and memory. These ROIs, which were 1.3–3 cm anterior-lateral to regions where previous studies had found word-category differences in brain activation in reading and listening tasks (see Results, cf.^73^), were contrasted. Activation in these regions was compared between the arm and leg word categories using repeated measures ANOVA.

## Results

### Behaviour

High accuracy rates (mean = 97.7%, standard error, SE = 0.3%) and d’ values (mean = 3.9, SE = 0.12) confirmed good performance in all subjects.

### Whole brain analysis

**Figure 2:**
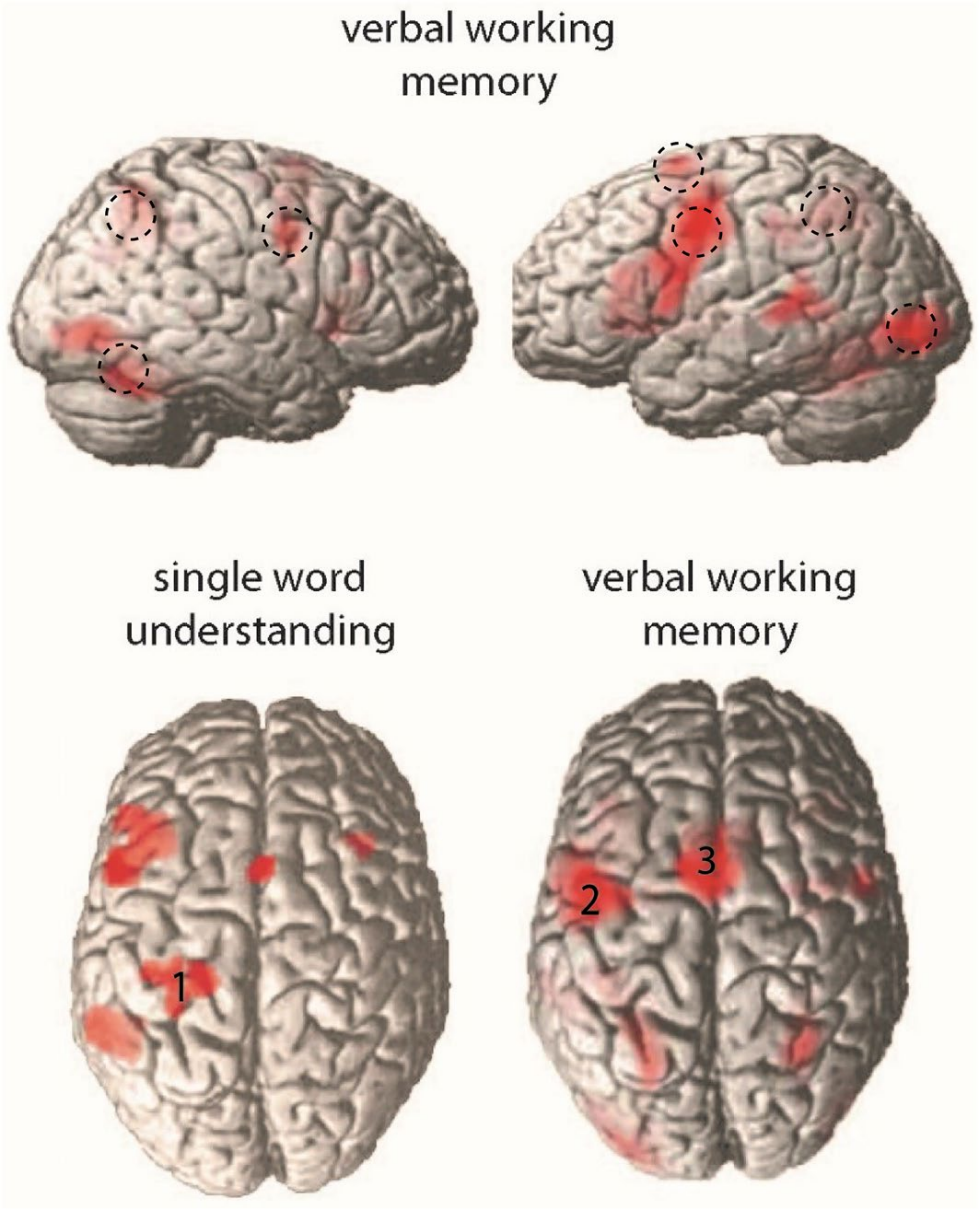
Top panels: Hemodynamic correlates of verbal memory load in the delayed nonmatching-to-sample task. Trials with high memory load are compared against a baseline of low memory load, while keeping constant both task and amount of stimulation. Both encoding and memory maintenance intervals are collapsed into this analysis. All clusters are significant at an FDR-corrected threshold *p* < 0.05. Bottom panels: Dorsal views of BOLD activation from a previous study^70^ during passive reading of action words (against a baseline of looking at matched meaningless symbol strings; bottom left) and of the memory load contrast (as in top panels). Note the central position of the activation focus labelled ‘1’ in sensorimotor cortex in the former and the more anterior foci labeled ‘2’ and ‘3’ in lateral and dorsomedial frontal cortex in the latter. Note also that the anterior left inferior prefrontal activation focus in the former (bottom left) is largely due to the face related words included in the study^70^, which were not used in the present study.

The memory load contrast (high load vs. low load), showed significant activation (*p* < 0.05, FDR corrected) in a range of areas (Table 2, Fig. 2, top and bottom right panels). One activated cluster appeared in left precentral gyrus (BA6), also extending to adjacent motor and prefrontal cortex (caudal BA8, 9). This cluster stretched from dorsolateral sites down into the posterior, premotor part of Broca’s area (BA44/45). A second cluster was in left dorsal supplementary motor area, SMA (BA6), and a third extended from left inferior-occipital cortex into inferior-temporal and superior-temporal sites. A fourth cluster in the left hemisphere included the intraparietal sulcus and adjacent temporal areas (BA 40, 7). Right hemispheric activation indicative of memory load was seen in precentral/prefrontal and parietal areas homotopic to the left-hemispheric ones. In addition, right inferior temporo-occipital cortex and possibly adjacent cerebellum showed general memory load effects.

**Table 2:** Significant areas of activation during encoding and memory (threshold at FDR 0.05).

Brain areas		Brodmann Areas	*p* value (FDR)	*t* value	Cluster size	MNI peak coordinates (mm)		
						x	y	z
Activations during encoding and memory								
1	L Precentral/prefrontal cortex	BA 6	< 0.001	13.08	8361	− 46	− 2	46
2	L Supplementary motor area	BA 6	< 0.001	10.47	2287	− 10	2	68
3	L Inferior occipital cortex	BA 19	< 0.001	8.22	3938	− 36	− 82	− 4
4	L Inferior parietal cortex	BA7/BA40	< 0.001	7.94	1694	− 26	− 56	42
5	R Inferior temporal-occipital cortex	BA37/BA19	0.001	6.80	2655	28	− 56	− 28
6	R Precentral gyrus	BA6	0.001	6.27	206	32	0	46
7	R Angular gyrus	BA7/BA40	0.003	5.15	1196	30	− 54	44

Peak coordinates in MNI space are listed using the Talairach coordinate system. *t* values are reported for magnitude of activation. Anatomical labels for peak coordinates were derived from the MRIcroN software. L, Left; R, Right.

**Figure 3:**
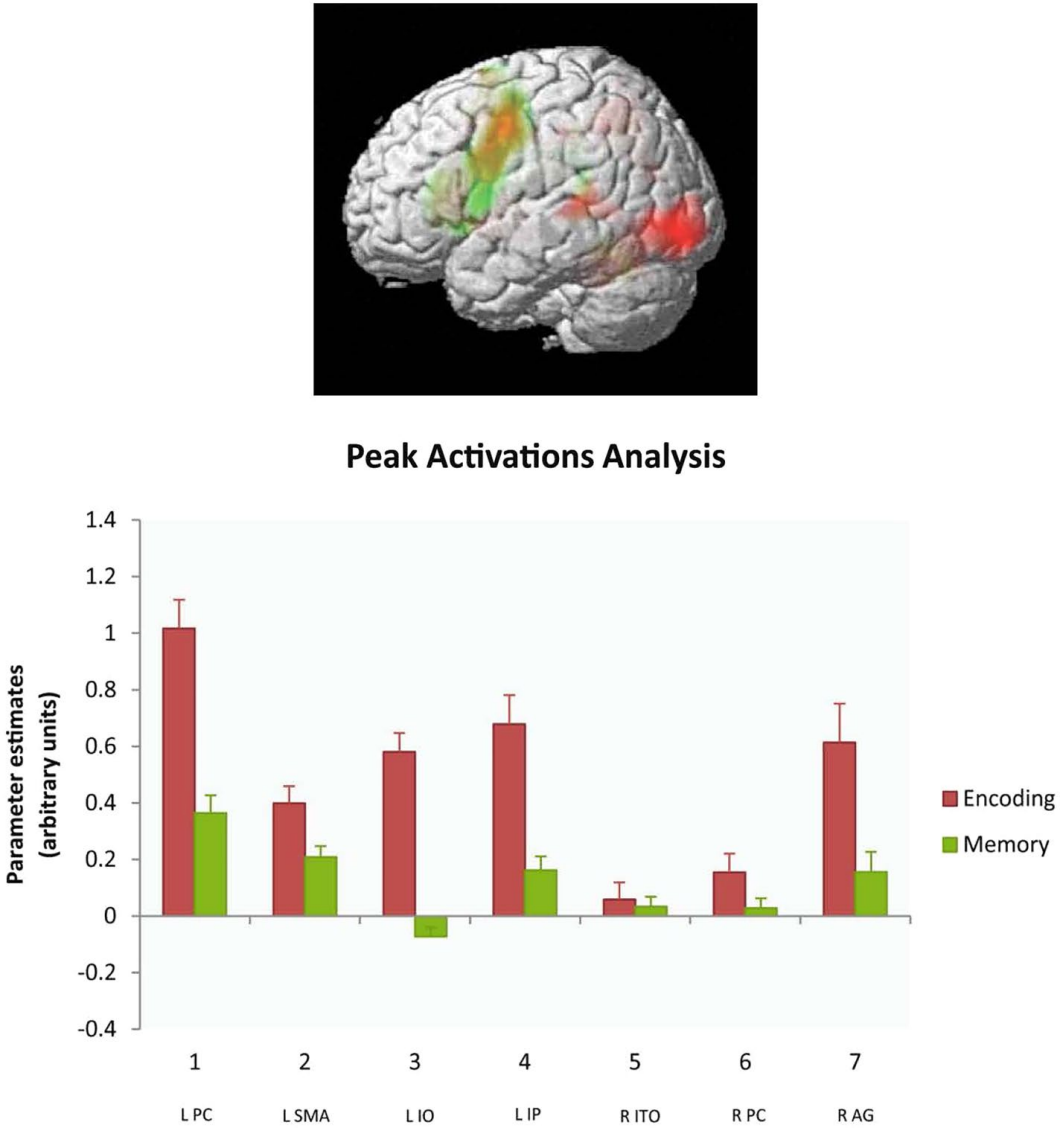
Memory load effects during the encoding interval (in red) contrasted with that during memory maintenance (in green; FDR *p* < 0.05). Note the pronounced overlapping activation in left dorsolateral premotor/prefrontal cortex and in the supplementary motor area. L PC, left precentral/prefrontal cortex; L SMA, left supplementary motor area; L IO, left inferior occipital cortex; L IP, left inferior parietal cortex; R ITO, right inferior temporal-occipital cortex; R PC, right precentral gyrus; R AG, right angular gyrus.

Whole brain analyses performed separately on the memory load contrasts obtained for the initial stimulus encoding interval and on those for the subsequent period of active memory maintenance indicated differences between these time periods. As Fig. 3 shows, stimulus encoding (in red) activated a range of areas, including temporo-occipital, superior-temporal and intra-parietal sites. Active memory maintenance (in green) produced activation in inferior-parietal and superior-temporal regions. A list of significant areas of activation for each time interval is presented in Table 3.

**Table 3:** Significant areas of activation during the encoding, memory and retrieval time intervals in the memory load contrast (threshold at FDR 0.05).

Brain areas	*p* value (FDR)	*t* value	Cluster size	MNI peak coordinates (mm)		
				x	y	z
**Activations during encoding**						
L Precentral gyrus	< 0.001	11.16	4887	− 46	− 2	46
L Inferior occipital cortex	< 0.001	9.87	3577	− 30	− 88	− 8
R Inferior occipital cortex	< 0.001	7.76	2645	48	− 76	− 6
L Supplementary motor area	< 0.001	7.69	1778	− 8	0	68
L Superior parietal cortex	< 0.001	7.28	1189	− 24	− 56	44
R Precentral gyrus	0.002	5.81	538	56	− 2	44
R Mid occipital	0.003	5.25	731	34	− 66	26
R Putamen	0.007	4.56	973	22	16	0
R Calcarine	0.022	3.53	36	16	− 70	10
R Mid frontal gyrus	0.026	3.42	32	40	28	22
**Activations during memory maintenance**						
L Inferior frontal gyrus (operculum)	0.014	7.95	8149	− 62	8	8
R Cerebellum	0.016	6.55	1626	24	− 60	− 24
L Supplementary motor area	0.018	5.61	1028	− 8	2	56
L Superior temporal cortex	0.024	4.62	126	− 52	− 42	18
L Inferior temporal cortex	0.027	4.24	201	− 42	− 46	− 12
R Caudate nucleus	0.028	4.08	57	18	28	10
R Insula	0.030	3.89	201	34	18	8
R Mid frontal	0.031	3.76	39	32	40	30
L Mid occipital cortex	0.034	3.59	105	− 24	− 58	42
**Activations during retrieval**						
R Superior Frontal gyrus	0.016	7.54	365	4	30	46
L Precentral gyrus	0.023	5.51	37	− 54	12	32

Peak coordinates in MNI space are listed using the Talairach coordinate system. *t* values are reported for magnitude of activation. Only cluster sizes > 30 are presented. Anatomical labels for peak coordinates were derived from the MRIcroN software and SPM 8. L, Left; R, Right.

### ROI analyses

#### ROI analysis 1

Somatotopic differences between memory load effects during encoding and active memory maintenance intervals was investigated using a data-driven analysis of regions of interest (ROIs), which were placed around the peak activation voxels of all FDR corrected clusters of the general high-versus-low-load contrast (Fig. 2, Table 2). Average activation values obtained for each of these ROIs in each time interval (encoding vs. maintenance) and word type (arm- vs. leg-related) were submitted to an analysis of variance, ANOVA (with factors ROI, time interval and word type), which revealed a significant interaction between the factors ROI and Interval (F (6,96) = 14,21, *p* < 0.00001). Significant differences between time intervals were confirmed by planned comparison F-tests in left prefrontal/premotor, parietal and temporo-occipital along with right parietal ROIs (Bonferroni-adjusted significance threshold: *p* < 0.014). These data-driven ROIs showed relatively stronger activation during memory encoding. During the memory maintenance interval, activation was primarily observed in premotor and SMA regions (Fig. 3).

**Figure 4:**
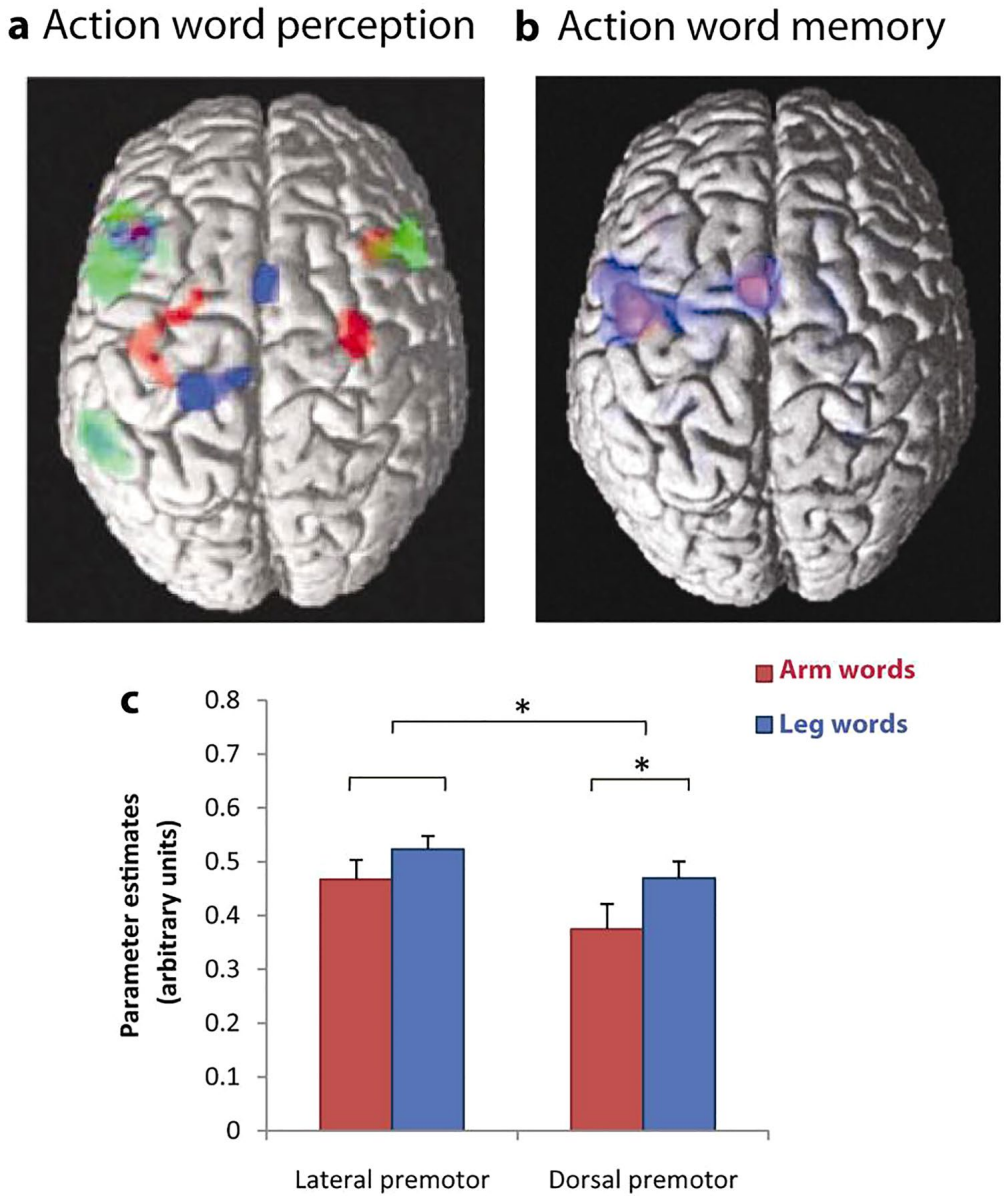
(**a**,**b**) Comparison between dorsal views of word category effects seen in the present working memory study and in an earlier study of word reading using a similar set of arm- and leg-related words^70^. The previous study’s results are displayed on the left, with activity to face-related words in green, that to arm-related words in red, and that to leg words in blue. The brain diagram on the right presents results on memory load effects from the present investigation (*p* < 0.001 uncorrected) with arm word-memory load highlighted in red and memory load for leg words in blue. Note the anterior shift of category-specific activation in verbal working memory relative to reading. (**c**) Significant interaction of ROI and Word Category in the present study showing stronger activation for leg-word than arm-word memory in dorsal premotor regions.

The data-driven ROI analysis did not provide evidence for brain activation differences between word types. However, when rendering memory-load effects for arm and leg words separately at an uncorrected threshold of *p* < 0.001 (Fig. 4), category differences were observed about 1–3 cm anteriorly to where action word related differences had been reported in previous studies^73,81^. Arm words (in red) activated inferior and lateral prefrontal and precentral areas, whereas leg words (in blue) activated additional more dorsal regions. Note that these tendencies towards differences were present not at the loci where strongest memory-load related activation was seen, but slightly anterior-lateral to these sites instead.

#### ROI analysis 2

In this analysis, in order to assess the hypothesis of an anterior shift of activation, two lateral ROIs (− 46 − 2 44, − 48− 4 50) were contrasted with two dorsal precentral ROIs (− 4 2 58, − 34 0 60). These ROIs were based on local activation maxima in frontocentral sensorimotor cortex. The ANOVA with the design ROI × Word Category on the parameter estimates averaged over the voxels in each pair of the lateral and dorsal ROIs revealed a significant interaction of ROI and Word Category (F (1, 16) = 9.79, *p* = 0.0065) due to stronger leg-word than arm-word memory activation in the dorsal regions (t (1, 16) = 1.85, *p* < 0.04, one-tailed), but no significant differences at lateral sites.

## Discussion

The aim of the present study was to examine the brain correlates of verbal working memory for action-related words. Specifically, we investigated whether action word memory maintenance would activate the same motor regions previously shown to be active during action word perception and understanding (as predicted by the embodied perspective), or the main regions associated with verbal working memory (as predicted by the Baddeley model with its emphasis on Broca’s region). As a third possibility, we considered the frontal memory shift hypothesis of current neurobiologically founded action perception theory, according to which active memory maintenance draws on multimodal connector hub areas and thus, in the case of action-related words, upon areas anterior to the motor regions previously found active during comprehension. Our results provide evidence for the anterior shift hypothesis and thus for the action perception model.

As an additional feature, we asked whether semantic differences between words related to actions typically performed with different parts of the body might lead to category-specific activations resembling the semantic somatotopy found in word reading or recognition experiments. The present results show memory load effects for arm- and leg-related action words in partially overlapping areas, with only weak evidence of category-specificity, anterior to the precentral sites previously associated with differences between semantic action word types. Please note once again that these results found in a memory task contrast with the previously reported pattern of activation during passive reading and listening to words. In these passive perceptual tasks, premotor and motor cortex showed meaning-related activation of arm and leg motor representations to arm- and leg-related words in a semantically somatotopic manner^5,68,82–86^. Such somatotopic motor systems activation was not present in our present data on memory maintenance. However, and interestingly, word category differences appeared in more anterior frontal regions than the previously reported category dissociations in motor systems. This observation strengthens the conclusion on an anterior frontal shift in verbal working memory.

To highlight the anterior shift of frontal activity during the present working memory task as compared with a passive reading and understanding paradigm, we contrast the results reported by Hauk et al.^70^ (their Fig. 1C, left panel) with the whole brain analysis of the present study (Fig. 2, bottom panels). It can be seen that, instead of a pronounced activation focus extending across the central sulcus (labeled by the number ‘1’), which was present in the word comprehension study, the current high versus low memory load contrast showed two significant foci, one encompassing lateral premotor and posterior prefrontal cortex and one including dorsomedial SMA (which are labeled ‘2’ and ‘3’). While comparing working memory effects to passive reading in the same participants would have been useful, comparing both types of processing in the same experiment was not possible due to time constraints and the complexity of the paradigm used. Contrasting the results of the two types of processing in Fig. 2, however, provides a visual comparison of how verbal working memory for action words elicited frontal activity in more anterior foci in the present study than the frontal activity found in the word reading task reported in Hauk et al.^70^.

### Working memory effects

Manipulating the load of verbal working memory, results of the whole brain analysis indicate that a distributed set of regions are important for memory encoding and maintenance. This network included regions well known to contribute to verbal working memory, especially left inferior-prefrontal/premotor and superior-temporal cortex, the cortical areas underpinning the articulatory and acoustic subparts of the phonological loop (Fig. 2)^37,87,88^. Baddeley’s model emphasizes the role of Broca’s region in verbal working memory. However, in the present results, the memory-active areas in the frontal lobe were not exhaustively described by the ‘Broca’s region’ label. The present results, therefore, cannot be fully accounted for by Baddeley’s model. A prefrontal-premotor lateral focus and a dorsomedial focus of activity in the present data was characteristic of the high versus low memory load contrast. The activation of these relatively anterior areas better fits the anterior shift prediction of the neurocomputational model of the dynamics of action perception circuits which we described in the introduction (see also Fig. 1).

In the present study, it was essential to choose a paradigm with high behavioral accuracy. A low proportion of errors was necessary to minimize the contribution of any error related brain responses (e.g. confounds due to different degrees of error generation, error detection and error correction). In order to make it very difficult for participants to engage in processes other than memory related ones, the task was made challenging in the high-load condition by rapid presentation of several semantically closely related items. During preliminary testing, the number of words that could be maintained in memory without behavioral errors was titrated, and it was found that, for the word material used in this study, a set of four was experienced as difficult by most subjects while at the same time still allowing accurate performance in the majority of trials (for behavioural results using the same stimuli, see^17^, but see also^18^). Accurate performance in a challenging task would require subjects to be fully engaged in the working memory task. Such a task would place high processing demand on neural resources to keep the semantically-related words in memory, making it difficult for subjects to engage in secondary activity such as imagery or other processes not related to memory maintenance. Furthermore, it would indeed not be helpful to apply a mnemonic strategy of visual imagery in the present context, for example to picture actions named by the verbal stimuli because, typically, several items with similar meaning were used (for example, walk, stroll, go, march or hop, jump, leap, skip), so that visual imagery would have led to underspecification of the to-be-memorised concepts, thus provoking verbal mistakes.

Results show that the topography of brain activation was modulated during the memory experiment. The encoding interval, during which stimuli were presented, showed strongest activation in premotor/prefrontal, left temporo-occipital and bilateral parietal areas. During memory maintenance, strongest activity was seen in left precentral and prefrontal, supplementary motor, left-perisylvian superior-temporal and bilateral parietal areas. The significant interaction of time interval by ROI in ROI Analysis 1 also indicates that the topography of brain activation was modulated. The fact that two premotor areas, prefrontal/premotor cortex and SMA, together dominate the neurometabolic memory load effect observed during the maintenance interval suggests a role of motor systems and adjacent prefrontal cortex in the maintenance of active verbal memories semantically linked to action. While peak activation was present in premotor cortex (− 46, − 2, 46), this activation equally involved adjacent dorsolateral prefrontal cortex (Fig. 3). The strong lateral and dorsal motor system activation seen in the present study is not commonly observed in studies of verbal working memory, where prefrontal and parietal activations together with the activation of perisylvian foci have been reported (see, for example,^36,53,88–90^). As an explanation of this pattern of activation during action word memory, it seems plausible to consider an influence of the semantics of the stimulus materials.

One might ask whether different load conditions had a differential impact on the delay activations and, specifically, whether stimulus encoding in the high load condition may have taken place in the beginning of the memory period. However, it is unlikely that there was any spill-over activation from the encoding phase. First, a variable delay period was used in the experiment in order to allow the disambiguation of the fMRI responses to the stimuli from those recorded during memory maintenance. Second, in the experiment, stimulus presentation terminated before the memory period started and numerous studies show that word recognition and encoding happen very quickly, within ca. 100–200 ms after stimulus onset (e.g.^91–93^). It is, therefore, unlikely that activation during recognition and encoding leaked into the memory period.

### Semantic somatotopy and verbal working memory: towards neuromechanistic integration

In the present experiment, memory load effects obtained for different action-related word categories did not appear in loci where embodied motor processes for action-related words emerged in a range of previous neuroimaging experiments^73,81,94^. Nor did they emerge at the primary motor cortex loci where TMS pulses had elicited causal effects on action word recognition which depended on the meaning of these items^95^. Instead, such category differences appeared when additional ROIs were defined around relative maxima of the hemodynamic load activations. These additional regions (lateral: − 46 − 2 44, − 48 − 4 50; dorsal: − 4 2 58, − 34 0 60) were 13–30 mm anterior to where previous studies using similar sets of stimuli (for an overview, see^73,81^) found differences in motor systems activations to words typically used to refer to arm and leg driven actions (lateral: − 38 − 20 48, dorsal: − 20 − 30 64,^70^). At these loci, a degree of category specificity emerged in the form of a significant interaction of the factors word type and region (dorsal vs. lateral), indicating that the working memory load effect is more pronounced for leg-related action words at these dorsal premotor sites than for arm-related words. This effect was significant for dorsal regions only. The lack of a similar significant effect in lateral sites could possibly be related to the task requirements. Although care was taken to reduce the amount of motor activation in the dominant left hemisphere, the occasional button presses as well as the inhibition of button presses in non-response trials may have obscured effects in the lateral region of interest, which is close to the hand motor representation. In summary, the memory task seemed to elicit significantly more anterior frontal activation compared with reading and listening tasks and the trace of category specificity in the memory load effects appeared at a somewhat ‘disembodied’ location, with substantial anterior shift.

Recent neurocomputational modelling work may provide an explanation for our present findings. An anterior shift of activation together with reduced topographic specificity are consistent with predictions of a neurocomputational model of action perception circuits, APCs, carrying word comprehension and verbal memory processes^60,96,97^. In this brain model mimicking frontal-temporal-occipital lobes^60^, the momentary full ignition of an APC spiking activity corresponds to the recognition and semantic understanding of a single word, whereas the subsequent reverberant (sustained) activity of the circuit is the material basis of verbal working memory^53^. Although the same action perception circuit which connects phonological knowledge about the word form in the perisylvian language areas with that about its meaning in modality preferential cortices, including grounding information about possible referents, is active in both comprehension and memory maintenance, different parts of the circuit across the distinct cortical areas are respectively active. Whereas all circuit parts partake in the ignition process, reverberation depends critically on the most strongly connected circuit parts that remained active for several time steps after full ignition, which implements memory maintenance^53^. Focusing on those network parts in frontal cortex, this leads to an anterior shift of the center of gravity of activity from motor to prefrontal cortices (Fig. 1, see blue pixels). Our present fMRI results support such anterior shift, which was revealed by both the general memory load contrast in the whole brain analysis as well as the observed traces of word category specific activations in ROI Analysis 2. The neurobiological explanation for such retreat of activity to prefrontal multimodal areas lies in the connectivity structure of the underlying neuroanatomical substrate of the connector hubs and their central status bridging between sensory and motor areas.

In the discussion about embodied cognition and the role of disembodied processes relying on multimodal areas, the present results offer an integrated perspective based on neurobiologically realistic APCs. These circuits emerge from correlated neuronal firing related to perceptions and bodily actions. Still, they encompass modality referential sensory and motor areas as well as multimodal connector hubs. They provide a mechanism both for the ‘embodied’ grounding of word forms in the real-world entities these symbols are used to speak about and for the ‘disembodied’ retreat of memory related neuronal activity to multimodal connector hub areas. As correlations in sensory and motor information are the major driving force in the formation of APCs, this account is largely consistent with principle ideas governing the embodied cognition framework. It is important to note this, as a simplistic version of an embodied cognition approach—which would postulate the same mechanisms to be equally relevant for language understanding and verbal memory, would clearly be falsified by the present data (for discussion of oversimplified embodied positions, see, for example,^98^). On the other hand, a pure disembodiment perspective situating semantics exclusively in multimodal areas does not even begin to offer an explanation for the present and related results.

The mechanism underpinning the anterior cognitive shift seen in the present study, therefore, may be one of disembodiment by which the activation of a distributed action-perception circuit dynamically moves from its sensorimotor periphery and focuses on its core parts in areas that link together action and perception systems, especially in PFC. Such neurofunctional disembodiment still allows action-perception circuits to mediate between memories, actions and perceptions, so that concurrent motor movements and their causal influence on working memory^11,17^ are also compatible with, and strongly predicted by, this model. PFC may therefore be a key component in this dynamic progression of memory activity because of its strong corticocortical connectivity to both action and perception systems of the brain. Support for our interpretation also comes from recent neuroimaging evidence suggesting that the PFC is a key region for the representation of fine-grained semantic similarities among words, across categories of action-related verbs and nouns^99^. Further integrating mechanisms of embodiment and disembodiment in perception, comprehension and working memory will be a challenging and exciting topic for future research into brain-grounded cognition.

## Conclusion

Our results show that verbal working memory for action-related words involves premotor areas, providing support for grounded and embodied theories of action-perception circuits in language and conceptual processing^1,2,4,6,29,100^. On the other hand, disembodiment was also evident in the brain responses, especially during the working memory maintenance interval, where activation was seen not at the somatotopic central loci where previous studies had found action word elicited activity, but rather in areas anteriolateral and dorsomedial to these regions, with only a trace of semantic somatotopy. This is evidence for an anterior cognitive shift in frontal cortex, possibly indicating progression from recognition and comprehension related ignition processes to reverberant memory activation within structured action-perception circuits. Even though the formation of these circuits appears to be driven by sensorimotor information, their core parts may lie in connector hubs or convergence zones of higher association cortex, including PFC, so that, during memory intervals, reverberation may gradually focus on these core parts. The results provide an important lead for neurocomputational studies that integrate memory disembodiment with current neuromechanistic theories rooted in action and perception.

## Supplementary Information

Supplementary Information 1.

Supplementary Information 2.

## Data Availability

The datasets generated during and/or analysed during the current study are available from the corresponding author on reasonable request.

## References

[1] Barsalou LW (2008). Grounded cognition. Annu. Rev. Psychol..

[2] Kiefer M, Pulvermüller F (2012). Conceptual representations in mind and brain: Theoretical developments, current evidence and future directions. Cortex.

[3] Willems RM, Casasanto D (2011). Flexibility in embodied language understanding. Front. Psychol..

[4] Pulvermüller F, Fadiga L (2010). Active perception: Sensorimotor circuits as a cortical basis for language. Nat. Rev. Neurosci..

[5] Hauk O, Pulvermüller F (2004). Neurophysiological distinction of action words in the fronto-central cortex. Hum. Brain Mapp..

[6] Glenberg AM, Gallese V (2012). Action-based language: A theory of language acquisition, comprehension, and production. Cortex.

[7] Klepp A (2014). Neuromagnetic hand and foot motor sources recruited during action verb processing. Brain Lang..

[8] Pulvermüller F, Hauk O, Nikulin VV, Ilmoniemi RJ (2005). Functional links between motor and language systems. Eur. J. Neurosci..

[9] Gerfo EL (2008). The influence of rTMS over prefrontal and motor areas in a morphological task: Grammatical vs. semantic effects. Neuropsychologia.

[10] Willems RM, Labruna L, D'Esposita M, Ivry R, Casasanto D (2011). A functional role for the motor system in language understanding: Evidence from theta-burst transcranial magnetic stimulation. Psychol. Sci..

[11] Shebani Z, Pulvermüller F (2018). Flexibility in language action interaction: The influence of movement type. Front. Hum. Neurosci..

[12] Vukovik N, Feurra M, Shpektor A, Myachykov A, Shtyrov Y (2017). Primary motor cortex functionally contributes to language comprehension: An online rTMS study. Neuropsychologia.

[13] Repetto C, Colombo B, Cipresso P, Riva G (2013). The effects of rTMS over the primary motor cortex: The link between action and language. Neuropsychologia.

[14] Gianelli C, Dalla Volta R (2015). Does listening to action-related sentences modulate the activity of the motor system? Replication of a combined TMS and behavioral study. Front. Psychol..

[15] Glenberg AM, Kaschak MP (2002). Grounding language in action. Psychon. Bull. Rev..

[16] Glenberg AM, Sato M, Cattaneo L (2008). Use-induced motor plasticity affects the processing of abstract and concrete language. Curr. Biol..

[17] Shebani Z, Pulvermüller F (2013). Moving the hands and feet specifically impairs working memory for arm- and leg-related action words. Cortex.

[18] Montero-Melis G, Van Paridon J, Ostarek M, Bylund E (2022). No evidence for embodiment: The motor system is not needed to keep action verbs in working memory. Cortex.

[19] Boulenger V (2006). Cross-talk between language processes and overt motor behavior in the first 200 msec of processing. J. Cogn. Neurosci..

[20] de Vega M, Moreno V, Castillo D (2013). The comprehension of action-related sentences may cause interference rather than facilitation on matching actions. Psychol. Res..

[21] Bak T, Chandran S (2012). What wires together dies together: Verbs, actions and neurodegeneration in motor neuron disease. Cortex.

[22] Bak T, O'Donovan DG, Xuereb JH, Boniface S, Hodges JR (2001). Selective impairment of verb processing associated with pathological changes in Brodmann areas 44 and 45 in the Motor Neurone Disease-Dementia-Aphasia syndrome. Brain.

[23] Boulenger V (2008). Word processing in Parkinson's disease is impaired for action verbs but not for concrete nouns. Neuropsychologia.

[24] Cotelli M (2006). Action and object naming in frontotemporal dementia, progressive supranuclear palsy and corticobasal degeneration. Neuropsychology.

[25] Kemmerer D, Rudrauf D, Manzel K, Tranel D (2012). Behavioural patterns and lesion sites associated with impaired processing of lexical and conceptual knowledge of action. Cortex.

[26] Fernandino L (2013). Parkinson’s disease disrupts both automatic and controlled processing of action verbs. Brain Lang..

[27] Vannuscorps G, Dricot L, Pillon A (2016). Persistent sparing of action conceptual processing in spite of increasing disorders of action production: A case against motor embodiment of action concepts. Cogn. Neuropsychol..

[28] Wurm MF, Caramazza A (2019). Distinct roles of temporal and frontoparietal cortex in representing actions across vision and language. Nat. Commun..

[29] Pulvermüller F (2013). How neurons make meaning: Brain mechanisms for embodied and abstract-symbolic semantics. Trends Cogn. Sci..

[30] Papeo L, Pascual-Leone A, Caramazza A (2013). Disrupting the brain to validate hypotheses on the neurobiology of language. Front. Hum. Neurosci..

[31] Mahon BZ, Caramazza A (2008). A critical look at the embodied cognition hypothesis and a new proposal for grounding conceptual content. J. Physiol. Paris.

[32] D'Esposito M (2007). From cognitive to neural models of working memory. Philos. Trans. R. Soc. B.

[33] Fuster JM (1995). Memory in the Cerebral Cortex: An Empirical Approach to Neural Networks in the Human and Nonhuman Primate.

[34] Baddeley A (1992). Working memory. Science.

[35] Baddeley A (2003). Working memory: Looking back and looking forward. Nat. Rev. Neurosci..

[36] Paulesu E, Frith CD, Frackowiak RS (1993). The neural correlates of the verbal component of working memory. Nature.

[37] Buchsbaum BR, D'Esposita M (2008). The search for the phonological store: From loop to convolution. J. Cogn. Neurosci..

[38] Schumacher EH (1996). PET evidence for an amodal verbal working memory system. Neuroimage.

[39] Cowan N (2005). Working Memory Capacity.

[40] Barrouillet P, Camos V, Osaka N, Logie R, D’Esposito M (2007). The time-based resource sharing model of working memory. The Cognitive Neuroscience of Working Memory.

[41] Camos V, Lagner P, Barrouillet P (2009). Two maintenance mechanisms of verbal information in working memory. J. Mem. Lang..

[42] Cowan N, Miyake A, Shah P (1999). An embedded-process model of working memory. Models of Working Memory: Mechanisms of Active Maintenance and Executive Control.

[43] Camos V, Mora G, Oberauer K (2011). Adaptive choice between articulatory rehearsal and attentional refreshing in verbal working memory. Mem. Cognit..

[44] Loaiza VM, Camos V (2018). The role of semantic representations in verbal working memory. J. Exp. Psychol. Learn. Mem. Cogn..

[45] Nishiyama R (2018). Separability of active semantic and phonological maintenance in verbal working memory. PLoS ONE.

[46] Shivde G, Anderson MC (2011). On the existence of semantic working memory: Evidence for direct semantic maintenance. J. Exp. Psychol. Learn. Mem. Cogn..

[47] Raye CL, Johnson MK, Mitchell KJ, Reeder JA, Greene EJ (2002). Neuroimaging a single thought: Dorsolateral PFC activity associated with refreshing just-activated information. Neuroimage.

[48] Raye CL, Johnson MK, Mitchell KJ, Greene EJ, Johnson MR (2007). Refreshing: A minimal executive function. Cortex.

[49] Rowe JB, Toni I, Josephs O, Frackowiak RS, Passingham RE (2000). The prefrontal cortex: Response selection or maintenance within working memory?. Science.

[50] Fuster JM (1997). The Prefrontal Cortex: Anatomy, Physiology, and Neuropsychology of the Frontal Lobes.

[51] Fuster JM (2015). The Prefrontal Cortex.

[52] Petrides M (2000). Dissociable roles of mid-dorsolateral prefrontal and anterior inferotemporal cortex in visual working memory. J. Neurosci..

[53] Fuster JM (2009). Cortex and memory: Emergence of a new paradigm. J. Cogn. Neurosci..

[54] Rowe JB (2007). Is the prefrontal cortex necessary for establishing cognitive sets?. J. Neurosci..

[55] Fuster JM (2003). Cortex and Mind: Unifying Cognition.

[56] Garagnani M, Wennekers T, Pulvermüller F (2008). A neuroanatomically grounded Hebbian-learning model of attention-language interactions in the human brain. Eur. J. Neurosci..

[57] Pulvermüller F (2018). Neural reuse of action perception circuits for language, concepts and communication. Prog. Neurobiol..

[58] Garagnani M, Pulvermüller F (2016). Conceptual grounding of language in action and perception: A neurocomputational model of the emergence of category specificity and semantic hubs. Eur. J. Neurosci..

[59] Tomasello R, Garagnani M, Wennekers T, Pulvermüller F (2017). Brain connections of words, perceptions and actions: A neurobiological model of spatio-temporal semantic activation in the human cortex. Neuropsychologia.

[60] Tomasello R, Garagnani M, Wennekers T, Pulvermüller F (2018). A neurobiologically constrained cortex model of semantic grounding with spiking neurons and brain-like connectivity. Front. Comput. Neurosci..

[61] Henningsen-Schomers MR, Pulvermüller F (2021). Modelling concrete and abstract concepts using brain-constrained deep neural networks. Psychol. Res..

[62] Tomasello M, Kruger AC (1992). Joint attention on actions: Acquiring verbs in ostensive and non-ostensive contexts. J. Child Lang..

[63] Pulvermüller F, Tomasello R, Henningsen-Schomers MR, Wennekers T (2021). Biological constraints on neural network models of cognitive function. Nat. Rev. Neurosci..

[64] Braitenberg V, Heim R, Palm G (1978). Cell assemblies in the cerebral cortex. Theoretical Approaches to Complex Systems (Lecture Notes in Biomathematics, vol 21).

[65] Moutard C, Dehaene S, Malach R (2015). Spontaneous fluctuations and non-linear ignitions: Two dynamic faces of cortical recurrent loops. Neuron.

[66] Palm G, Knoblauch A, Hauser F, Schuz A (2014). Cell assemblies in the cerebral cortex. Biol. Cybern..

[67] Pulvermüller F, Shtyrov Y, Ilmoniemi RJ (2005). Brain signatures of meaning access in action word recognition. J. Cogn. Neurosci..

[68] Shtyrov Y, Butorina A, Nikolaeva A, Stroganova T (2014). Automatic ultrarapid activation and inhibition of cortical motor systems in spoken word comprehension. Proc. Natl. Acad. Sci. U.S.A..

[69] van den Heuvel MP, Sporns O (2013). Network hubs in the human brain. Trends Cogn. Sci..

[70] Hauk O, Johnsrude I, Pulvermüller F (2004). Somatotopic representation of action words in the motor and premotor cortex. Neuron.

[71] Martin A, Wiggs C, Ungerleider L, Haxby J (1996). Neural correlates of category-specific knowledge. Nature.

[72] Martin A (2007). The representation of object concepts in the brain. Annu. Rev. Psychol..

[73] Carota F, Moseley R, Pulvermüller F (2012). Body-part specific representations of semantic noun categories. J. Cogn. Neurosci..

[74] Pecher D (2013). No role for motor affordances in visual working memory. J. Exp. Psychol. Learn. Mem. Cogn..

[75] Pecher D (2013). The role of affordances for working memory for objects. J. Cogn. Psychol..

[76] Oldfield RC (1971). The assessment and analysis of handedness: The Edinburgh inventory. Neuropsychologia.

[77] Smith SM (2002). Fast robust automated brain extraction. Hum. Brain Mapp..

[78] Maes F, Collignon A, Vandermeulen D, Marchal G, Suetens P (1997). Multimodality image registration by maximization of mutual information. IEEE Trans. Med. Imag..

[79] Friston KJ (1998). Event-related fMRI: Characterizing differential responses. Neuroimage.

[80] Tzourio-Mazoyer N (2002). Automated anatomical labeling of activations in SPM using a macroscopic anatomical parcellation of the MNI MRI signle-subject brain. Neuroimage.

[81] Kemmerer D, Gonzalez-Castillo J (2010). The two-level theory of verb meaning: An approach to integrating the semantics of action with the mirror neuron system. Brain Lang..

[82] Pulvermüller F, Hummel F, Harle M (2001). Walking or talking? Behavioural and neurophysiological correlates of action verb processing. Brain Lang..

[83] Boulenger V, Hauk O, Pulvermüller F (2009). Grasping ideas with the motor system: Semantic somatotopy with idiom comprehension. Cereb. Cortex.

[84] Shtyrov Y, Hauk O, Pulvermüller F (2004). Distributed neuronal networks for encoding category-specific semantic information: The mismatch negativity to action words. Eur. J. Neurosci..

[85] Kemmerer D, Castillo JG, Talavage T, Patterson S, Wiley C (2008). Neuroanatomical distribution of five semantic components of verbs: Evidence from fMRI. Brain Lang..

[86] Grisoni L, Dreyer FR, Pulvermüller F (2016). Somatotopic semantic priming and prediction in the motor system. Cereb. Cortex.

[87] Thierry G, Ibarrola D, Demonet JF, Cardebat D (2003). Demand on verbal working memory delays haemodynamic response in the inferior prefrontal cortex. Hum. Brain Mapp..

[88] Sakai K, Passingham RE (2003). Prefrontal interactions reflect future task operations. Nat. Neurosci..

[89] Barde LH, Thomspson-Schill SL (2002). Models of functional organization of the lateral prefrontal cortex in verbal working memory: Evidence in favor of the process model. J. Cogn. Neurosci..

[90] Jha AP, McCarthy G (2000). The influence of memory load upon delay-interval activity in a working-memory task: An event-related functional MRI study. J. Cogn. Neurosci..

[91] Amsel BD, Urbach TP, Kutas M (2013). Alive and grasping: Stable and rapid semantic access to an object category but no object graspability. Neuroimage.

[92] Pulvermüller F, Shtyrov Y, Hauk O (2009). Understanding in an instant: Neurophysiological evidence for mechanistic language circuits in the brain. Brain Lang..

[93] Hauk O, Coutout C, Holden A, Chen Y (2012). The time-course of single-word reading: Evidence from fast behavioral and brain responses. Neuroimage.

[94] Kemmerer D (2015). Are the motor features of verb meanings represented in the precentral motor cortices? Yes, but within the context of a flexible, multilevel architecture for conceptual knowledge. Psychon. Bull. Rev..

[95] Pulvermüller F (2005). Brain mechanisms linking language and action. Nat. Rev. Neurosci..

[96] Pulvermüller F, Garagnani M (2014). From sensorimotor learning to memory cells in prefrontal and temporal association cortex: A neurocomputational study of disembodiment. Cortex.

[97] Garagnani M, Pulvermüller F (2013). Neuronal correlates of decisions to speak and act: Spontaneous emergence and dynamic topographies in a computational model of frontal and temporal areas. Brain Lang..

[98] Barsalou LW (2016). On staying grounded and avoiding quixotic dead ends. Psychon. Bull Rev..

[99] Carota F, Kriegeskorte N, Nili H, Pulvermüller F (2017). Representational similarity mapping of distributional semantics in left inferior frontal, middle temporal, and motor cortex. Cereb. Cortex.

[100] Barsalou LW (1999). Perceptual symbol systems. Bahav. Brain Sci..

[101] Shebani Z (2018). Brain correlates of action word memory. BioRxiv.

