# Supplementary material for: Brain correlates of action word memory revealed by fMRI
Source: Sci Rep. 2022 Sep 26;12:16053. doi: 10.1038/s41598-022-19416-w (PMC9512810; doi:10.1038/s41598-022-19416-w)
Supplement: Supplementary file 1 — Supplementary Information 1. [file 41598_2022_19416_MOESM1_ESM.docx]

**Supplementary Figure S1 Caption**

Regions of interest in ROI Analyses 1 and 2. Left: Activation differences between memory load effects for arm and leg related action words during encoding and memory maintenance. ROIs were placed around the peak activation voxels of all FDR corrected clusters of the high-vs.-low-load contrast in the whole brain analysis. Right: Activation differences to arm and leg related action words in two lateral ROIs and two dorsal precentral ROIs. ROIs were selected based on local activation maxima in frontocentral sensorimotor cortex during encoding and memory. Numbers indicate z coordinates in MNI space.
