# Supplementary figures and images for: Brain correlates of action word memory revealed by fMRI
Source: Sci Rep. 2022 Sep 26;12:16053. doi: 10.1038/s41598-022-19416-w (PMC9512810; doi:10.1038/s41598-022-19416-w)

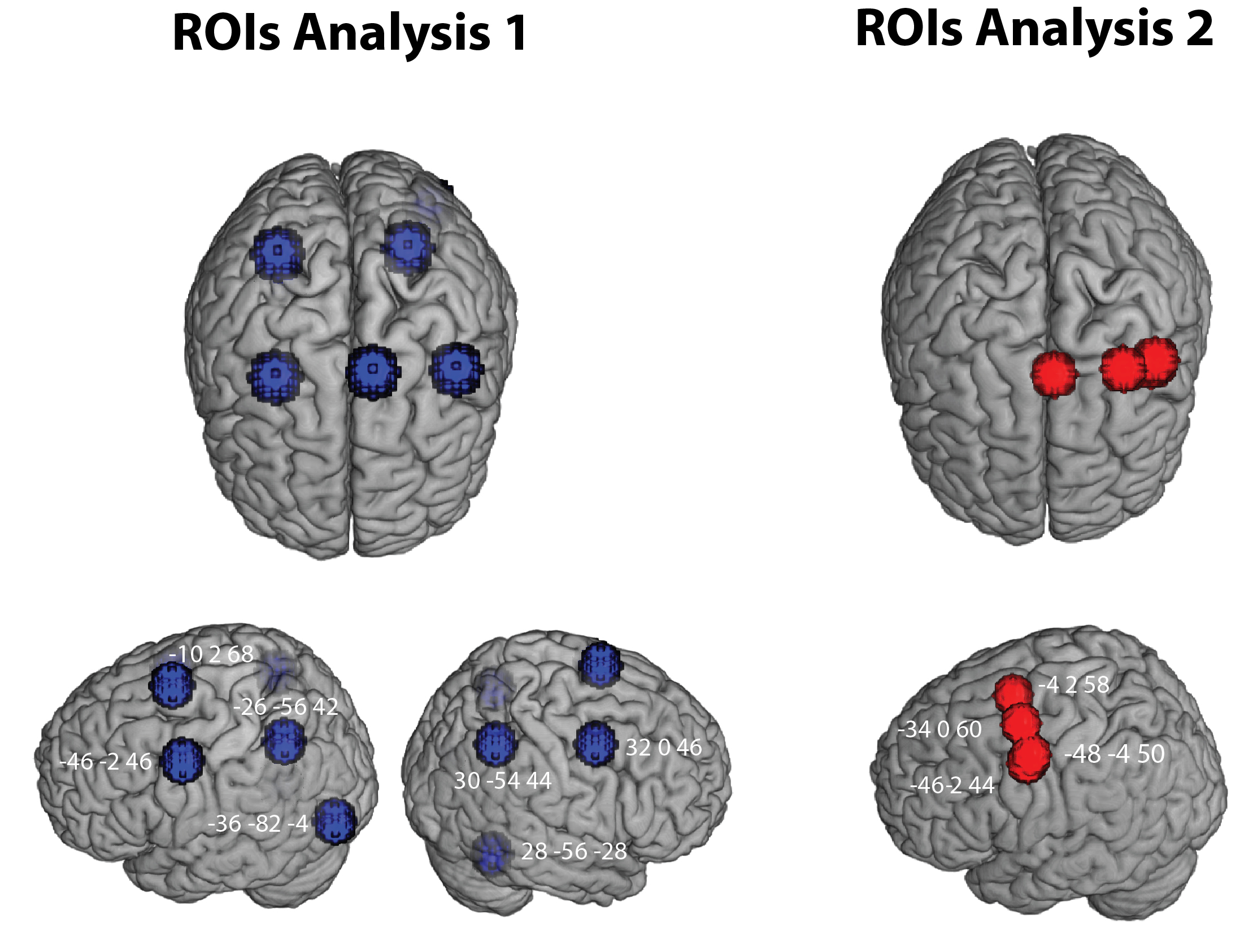

Supplement: Supplementary file 2 — Supplementary Information 2. [file 41598_2022_19416_MOESM2_ESM.png]
